# USP51 promotes non-small cell lung carcinoma cell stemness by deubiquitinating TWIST1

**DOI:** 10.1186/s12967-023-04304-2

**Published:** 2023-07-08

**Authors:** Jin Chen, Zhongqiu Wu, Wenyi Deng, Minying Tang, Lvying Wu, Na Lin, Liuyan Chen, Yunfeng Fu, Min Zhao, Changguo Chen, Wenting Li

**Affiliations:** 1 Institute of Clinical Medicine, The Second Affiliated Hospital of Hainan Medical University, Haikou, China; 2 Department of Clinical Laboratory, The Second Affiliated Hospital of Hainan Medical University, Haikou, China; 3 Fujian Provincial Key Laboratory of Transplant Biology, 900th Hospital, Xiamen University, Xiamen, China; 4 Department of Ultrasound, 900th Hospital, Xiamen University, Xiamen, China; 5 Department of Clinical Laboratory, The Sixth Medical Center of PLA General Hospital, Beijing, China; 6 Department of Infectious and Tropical Diseases, The Second Affiliated Hospital of Hainan Medical University, Haikou, China

**Keywords:** USP51, TWIST1, Non-small cell lung carcinoma, Deubiquitinase, Cell stemness

## Abstract

**Background:**

USP51 is a deubiquitinase (DUB), that is involved in diverse cellular processes. Accumulating evidence has demonstrated that USP51 contributes to cancer development. However, its impact on non-small cell lung carcinoma (NSCLC) cell malignancy is largely unknown.

**Methods:**

In this study, we performed bioinformatics analysis on a dataset from The Cancer Genome Atlas to determine the association between USP51 and cell stemness marker expression in NSCLC patients. RT‒qPCR, Western blotting, and flow cytometry were performed to examine the effects of USP51 depletion on stemness marker expression. Colony formation and tumor sphere formation assays were used to assess the stemness of NSCLC cells. A cycloheximide chase time-course assay and a polyubiquitination assay were carried out to analyze the effects of USP51 on the TWIST1 protein level. TWIST1 was overexpressed in USP51 knockdown NSCLC cells to determine whether TWIST1 is required. The effect of USP51 on the in vivo growth of NSCLC cells was tested through subcutaneous injections in mice.

**Results:**

We found that USP51 deubiquitinates TWIST1, which is significantly upregulated in the tissues of patients with NSCLC and is closely associated with poor prognosis. USP51 expression was positively correlated with the expression of stemness marker CD44, SOX2, NANOG, and OCT4 in NSCLC patients. USP51 depletion attenuated mRNA, protein, and cell surface expression of stemness markers and the stemness of NSCLC cells. Ectopic USP51 expression potentiated the stability of the TWIST1 protein by attenuating its polyubiquitination. In addition, TWIST1 re-expression in NSCLC cells reversed the inhibitory effect of USP51 knockdown on cell stemness. Furthermore, the in vivo results confirmed the suppressive effect of USP51 depletion on NSCLC cell growth.

**Conclusions:**

Our results show that USP51 maintains the stemness of NSCLC cells by deubiquitinating TWIST1. Knocking it down reduces both cell stemness and growth of NSCLC cells.

**Supplementary Information:**

The online version contains supplementary material available at 10.1186/s12967-023-04304-2.

## Background

Despite the reduced incidence of lung carcinoma and resulting cancer-related deaths over the past 20 years, 27% of cancer-related deaths still result from lung carcinoma [1, 2]. Non-small cell lung carcinoma (NSCLC), a major type of lung carcinoma, accounts for 85% of all lung carcinoma cases [3]. Because of the investigation and application of multiple diagnostic and prognostic biomarkers for NSCLC, the 5-year survival rate of patients with NSCLC has increased significantly [4]. Evaluation of EGFR mutations, detected in 15% of NSCLC patients, has guided targeted therapeutic approaches for patients, leading to a longer survival times [5]. However, cumulative clinical observations have shown that a certain group of patients acquire resistance to drugs targeting EGFR during chemotherapy [5]. Therefore, unraveling the mechanism of NSCLC progression may aid in the development of more effective adjuvant therapeutic strategies to overcome drug resistance.

The self-renewal and differentiation capabilities of stem cells are fine-tuned during tissue development and maintenance [6]. Cancer stem cells, a small population of cancer cells that are able to self-renew during cancer progression, contribute greatly to chemoresistance and cancer recurrence [7]. Moreover, acquisition of stemness endows cancer cells with aggressiveness and the capability to survive in the circulatory system, which results in their seeding in distant organs and, eventually, the formation of metastases [7]. Thus, screening for drugs that specifically target cancer stem cells has emerged as a novel approach for developing treatments targeting malignant cancer cells [8, 9]. Furthermore, the identification of target genes that potentiate cancer cell stemness can facilitate the further development of therapeutic drugs.

Polyubiquitination is a critical process for controlling protein abundance at the posttranslational level [10]. With the help of E1 activating and E2 conjugating enzymes, certain E3 ligases can recognize and target proteins and tag them with polyubiquitin chains. These polyubiquitinated proteins are accumulated and degraded by proteasomes or lysosomes [11]. In contrast, deubiquitinases (DUBs) are able to cleave ubiquitin chains and thus function to reverse the effects of E3 ligase molecules to modulate protein stability by cleaving the ubiquitin chains from proteins and recycling the ubiquitin molecules [12]. E3 ligases and DUBs constitute a dynamically balanced system that regulates protein expression. A previous study demonstrated that TWIST1 increases stemness of lung cancer cells [13] and promotes the occurrence and progression of NSCLC [14]. However, limited research has been conducted on the mechanisms responsible for regulating TWIST1 stability in lung cancer. Further investigation is required to determine whether DUBs regulate TWIST1.

Here, we show that the expression of USP51, a DUB whose function has not been characterized in NSCLC, is positively associated with the expression of a panel of stemness markers. The results of loss-of-function studies suggested that USP51 knockdown reduced the expression of stemness markers in NSCLC cells, thereby diminishing the growth and tumor sphere formation of NSCLC cells. Mechanistically, USP51 interacted with and deubiquitinated TWIST1, leading to TWIST1 protein stabilization. Moreover, restoration of TWIST1 expression in NSCLC cells alleviated the biological effects of the loss of USP51. Our study identified USP51 as a novel promoter of NSCLC cell stemness and revealed an unexpected mechanism by which USP51 exerts its effects in NSCLC cells.

## Materials and methods

### Analysis of data from the Cancer Genome Atlas (TCGA)

Normalized fragments per kilobase of transcript per million mapped (FPKM) values, genetic alteration landscapes, and clinical data were acquired from TCGA through the R package TCGAbiolinks (v.2.24.3) [15]. In total, data for 1149 non-small cell lung cancer (NSCLC) samples, namely, 598 lung adenocarcinoma (LUAD;59 normal and 539 tumor tissue) samples and 551 lung squamous cell carcinoma (LUSC; 49 normal and 502 tumor tissue) samples, were obtained. Paired samples of tumor and normal tissue were available for only 107 of the NSCLC patients. The expression of *TWIST1* in various tumor tissues, in tissues from NSCLC tumors of different stages was analyzed. Survival analysis, which was performed using the R packages survival (v3.3-1) and survminer (v.0.4.9), was conducted in TCGA cohorts to evaluate the impact of gene expression on the overall survival (OS) of NSCLC patients. Spearman correlation analysis was performed on data in the Gene Expression Profiling Interactive Analysis 2 (GEPIA2, http://gepia2.cancer-pku.cn/#index) database to analyze the correlations between *USP51* expression and the expression of *CD44*, *SOX2, NANOG, OCT4, C-MYC, and EPCAM* in LUSC and LUAD tumor samples in the TCGA database.

### Analysis of data from Gene expression Omnibus (GEO)

Lung cancer patient data were downloaded from GEO (accession: GSE19804) and included clinical data (age and stage), and expression data for *TWIST1* (213943_at) and *USP51* (229278_at). There were 60 pairs of lung cancer tissue and matched paracancerous normal tissue. Twenty-nine patients were 60 years old, and 31 patients were more than 60 years old. Of the 60 patients, 16 had stage 1 A disease, 19 had stage 1B disease, 5 had stage 2 A disease, 7 had stage 2B disease, 8 had stage 3 A disease, 4 had stage 3B disease, and 1 had stage 4 disease. The expression of *USP51* and *TWIST1* was compared between normal and tumor tissues, between more than 60 years old and those 60 years old or younger, and between tumor tissues of different stages using GraphPad Prism (version 8).

### Cell culture

HCC827 and NCI-H1299 cells (IMMOCELL, China) were cultured in RPMI 1640 medium (Hyclone Cytiva, SH30027.02) supplemented with 10% fetal bovine serum (FBS; Gibco, 10099141 C). All cells were maintained at 37 °C in a humidified atmosphere containing 5% CO_2_.

### Screen of DUBs

HEK-293T cells, which were stably overexpressing TWIST1-GFP, were cultured in 96-well plates at a density of 3 × 10^4^ cells per well for a duration of 6 h. Subsequently, the plasmids encoding 40 different DUBs was transfected individually into the cells. Following a 48-h incubation period, fluorescence intensity was quantified using an Elispot immunSpot^®^ S5 UV analyzer (CTL).

### Plasmid construction

Ectopic *USP51* and *TWIST1* expression constructs were generated by PCR amplification and inserted into the pLV-EF1a-HA-IRES-bsd and pLV-EF1a-Flag-IRES-bsd backbones, respectively. *USP51* shRNA was synthesized by ligating the annealed shRNA oligo into the pLKO.1-TRC backbone. All the primers used for cloning are listed in Additional file 1: Table S1. The plasmid encoding His-ubiquitin was purchased from Xiamen Antihela Biotechnology Co., Ltd.

### Real-time quantitative PCR (RT‒qPCR)

Total RNA was extracted using an RNA isolation kit (Omega, USA) with a standard protocol. Subsequently, 2 µg of the extracted RNA was reverse transcribed using a RevertAid First Strand cDNA Synthesis Kit (Thermo Fisher, K1621). RT‒qPCR was performed using SYBR Green Master Mix (Roche, 4913914001) on an ABI 7500 system (ABI, USA). The primers are listed in Additional file 1: Table S1. Relative mRNA expression was calculated based on the 2^−ΔΔCt^ method. The expression of 18S rRNA was used as an internal control for normalization.

### Western blotting

Western blot analysis was performed as previously described [16]. In brief, cells were lysed with RIPA buffer (Beyotime, P0013B) and the protein concentration was determined using a BCA protein assay kit II (Abcam, ab287853). Proteins were transferred onto a PVDF membrane after separation by 10% SDS‒PAGE, and 5% skim milk was used to block nonspecific binding. After incubation with primary antibodies and horseradish peroxidase-conjugated secondary antibodies (Additional file 1: Table S2), signals were visualized by a ChemiDoc Imaging System (Bio-Rad).

### Colony formation assay

After transfection with the pLKO.1-TRC, pLKO.1-USP51-TRC, or pLV-EF1a-Flag-TWIST1-IRES-bsd plasmid using Lipofectamine™ 2000 (Invitrogen, 11668019) for 24 h, 1 × 10^3^ NSCLC cells were seeded into the wells of a 6-well plate. After approximately 14 days of subculture, the cells were washed once with PBS and immediately fixed with methanol. Fixed viable cells were stained with crystal violet by incubation at room temperature for 15 min. Finally, colonies were photographed and counted under a light microscope (Motic).

### Tumor sphere formation assay

A total of 1 × 10^3^ transfected NSCLC cells were seeded into 24-well plates and maintained in DMEM/F12 medium containing B27 supplement (Gibco, 17504044, USA), bFGF (20 ng/mL, Novoprotein, China), EGF (20 ng/mL, Novoprotein, China), and insulin (4 µg/mL, Gibco, USA). After 12–18 days of culture, the spheres were photographed under a light microscope.

### Flow cytometry assay

After transfection, cells were washed with PBS. After centrifugation at 300×*g* for 5 min, the cells were resuspended and subsequently incubated with FITC-conjugated mouse monoclonal antibodies against human CD44 and EPCAM or with isotype control antibodies (BD, USA). Signals were detected using a flow cytometer (FACScan, BD, USA). Unstained cells were used as a negative control to establish the threshold of background fluorescence. Three independent experiments were carried out.

### Coimmunoprecipitation (Co-IP)

HCC827 cells were transfected with pLKO.1-TRC, pLV-EF1a-HA-USP51-IRES-bsd, pLV-EF1a-Flag-TWIST1-IRES-bsd, and/or a plasmid encoding His-ubiquitin using Lipofectamine™ 2000 (Invitrogen, 11668019) for 24 h. Cells were harvested and lysed by immunoprecipitation buffer (150 mmol/L NaCl, 50 mmol/L Tris–HCl pH = 7.4, 40 mmol/L -glycerophosphate, 1 mmol/L NaV_4_O_3_, 10 mmol/L NaF, and 2 mmol/L EDTA), supplemented with 1 mmol/L PMSF and protease inhibitor. Protein A/G beads were incubated with an anti-Flag antibody (Proteintech, catalog number: 66008-4-Ig) at 4 °C overnight and were then incubated with cell lysates at 4 °C for 6 h. Immunoprecipitates were washed with immunoprecipitation buffer and then subjected to Western blotting.

### Animal experiments

Animal experiments were carried out following a protocol approved by the Animal Ethics Committee of Hainan Medical University (HYLL-2022-390). Briefly, 5 × 10^6^ HCC827 cells were subcutaneously injected into the lower dorsal surface of BALB/c nude mice (6 weeks old, male, n = 5). Beginning 22 days post-inoculation, the tumors were measured with calipers every 4 days, and the tumor volume was calculated according to the following formula: length × width^2^/2. Forty-two days after inoculation, the mice were euthanized, and the tumors were weighed and photographed.

### Statistical analyses

Statistical analyses were carried out using GraphPad Prism 8.0 software. One-way ANOVA with Tukey’s post hoc test was used for comparisons among multiple groups. Student’s t tests were used to determine the significance of differences between two groups. All quantitative data are presented as the means ± SDs. *p < 0.05, **p < 0.01, ***p < 0.001, ****p < 0.0001. ns, not significant.

## Results

### TWIST1 is upregulated in NSCLC patients and significantly correlated with poor prognosis

**Fig. 1:**
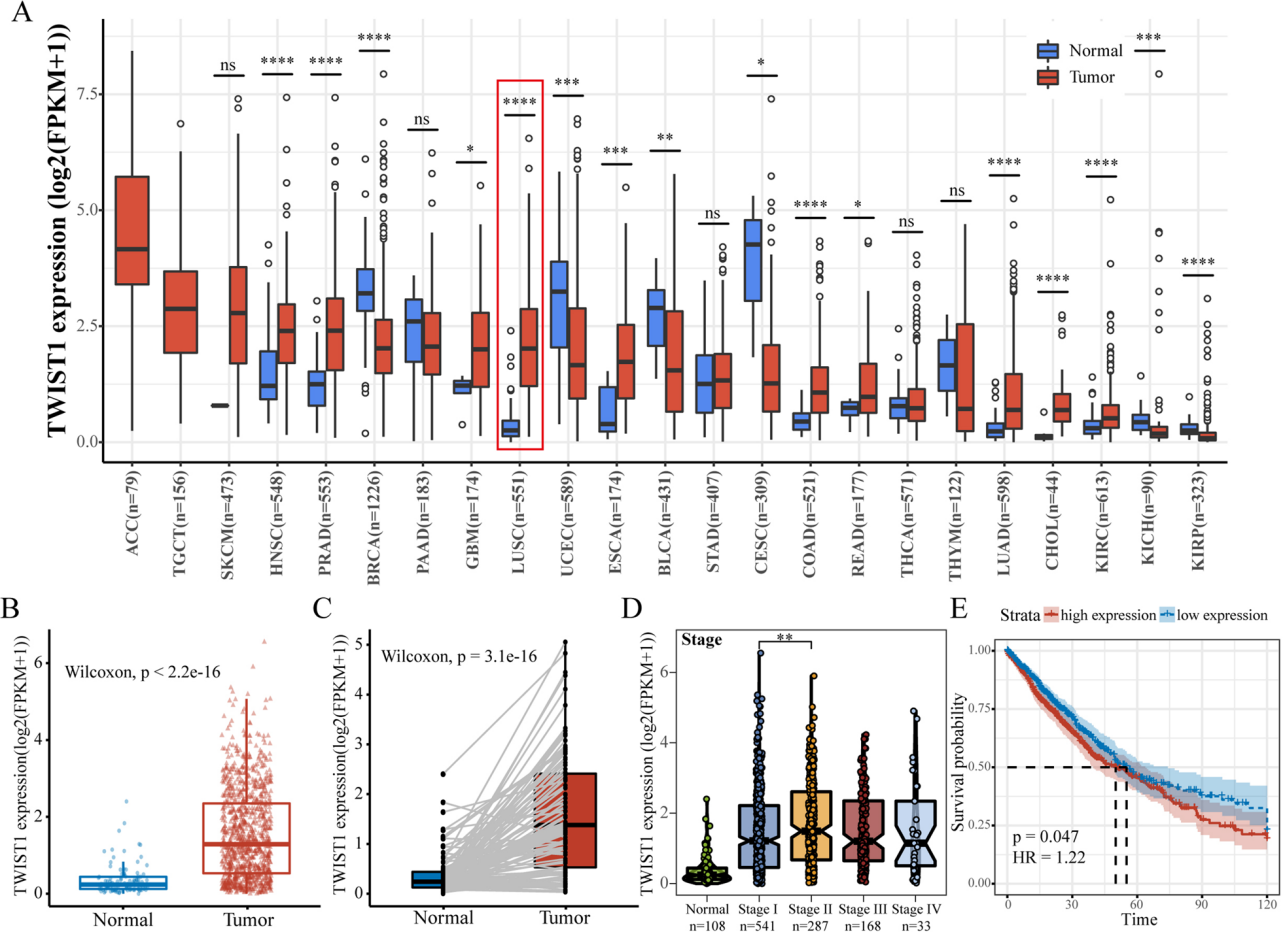
Analysis of *TWIST1* data from TCGA. **A** The expression of *TWIST1* in various tumors was compared with that in adjacent normal tissues. **B**, **C** The expression of TWIST1 in paired or unpaired NSCLC was compared with that in adjacent normal tissue. **D** The expression of TWIST1 was compared in NSCLC patients with different stages, (**E**) Kaplan–Meier survival curve of OS in NSCLC patients with high or low TWIST1 levels

The role of *TWIST1* in LUSC and LUAD were preliminarily explored through bioinformatics analysis. TCGA data showed that *TWIST1* was highly expressed in LUSC and LUAD (Fig. 1A–C). The *TWIST1* level was higher in stage II LUSC and LUAD tumors than in stage I LUSC and LUAD (Fig. 1D). Moreover, patients with high levels of *TWIST1* had worse overall survival outcomes than those with low levels (Fig. 1E). Overall, these finding indicate that *TWIST1* plays an important role in the development of LUSC and LUAD.

### USP51 binds to TWIST1, and its expression correlates with stemness marker expression in NSCLC patients

**Fig. 2:**
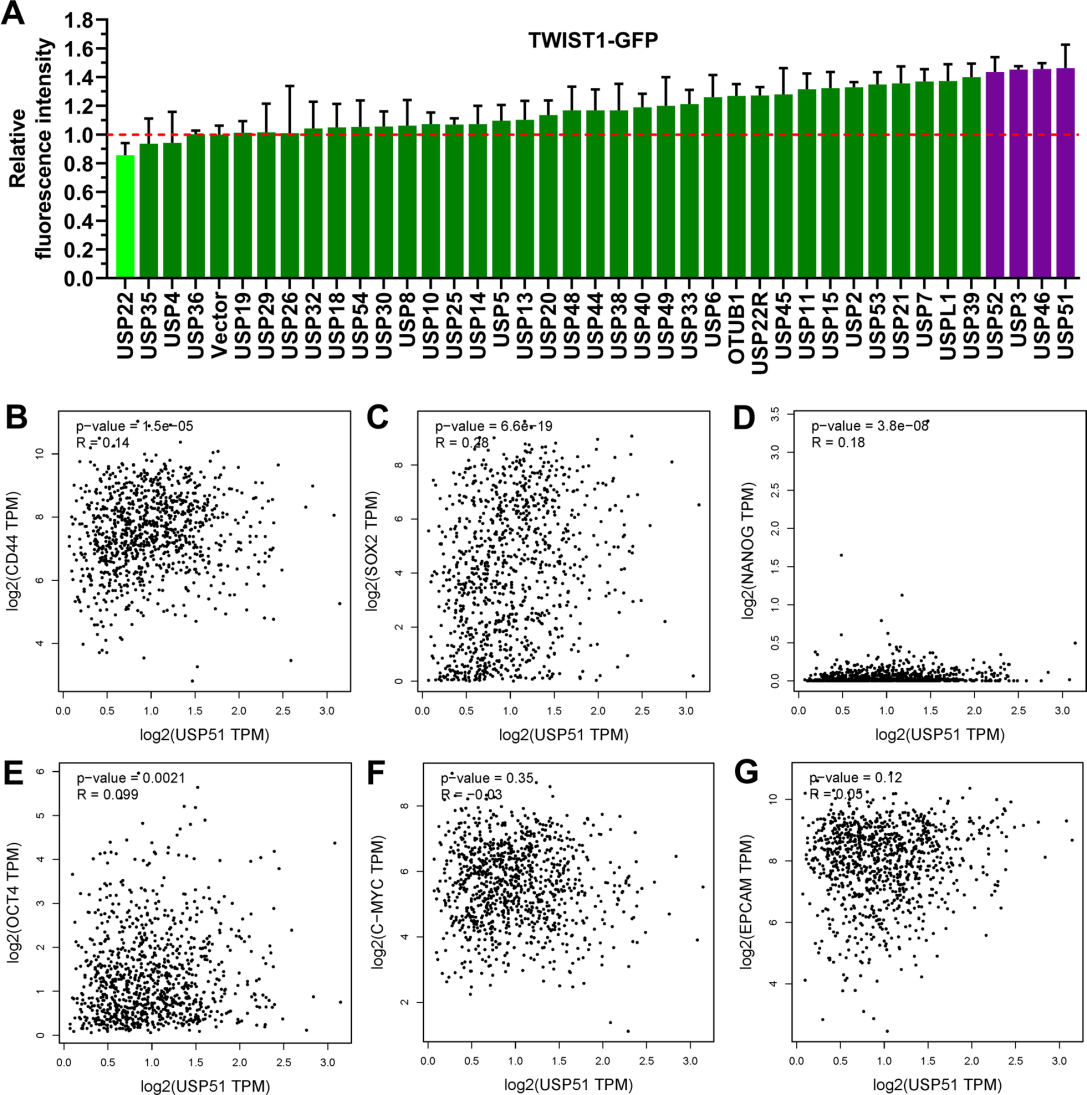
USP51 binds to TWIST1 and shows a positive correlation with the expression of stem cell markers in NSCLC patient cohorts from TCGA. **A** Statistical analysis of the fluorescence intensity of overexpressing individual DUBs in TWIST1-GFP stably overexpressed HEK-293T cells. **B**–**G** Scatter plots showing the correlations between the USP51 expression level and the expression levels of stemness markers, including *CD44* (**B**), *SOX2* (**C**), *NANOG* (**D**), *OCT4* (**E**), *C-MYC* (**F**) and *EPCAM* (**G**) by Spearman correlation analyses

Forty DUBs were screened in TWIST1-GFP stably overexpressed HEK-293T cells, and the cells transfected with USP51 demonstrated the strongest fluorescence signal (Fig. 2A). This suggests that USP51 has the potential to increase the expression of TWIST1 protein. Furthermore, we analyzed data from GEO (GSE19804) and found that *TWIST1* (213943_at) and *USP51* (229278_at) were both highly expressed in lung cancer tissues (Additional file 1: Fig. S1A, B). These findings further suggest that *TWIST1* expression is positively correlated with *USP51* expression in lung cancer. Further, we analyzed the correlation between *USP51* expression and the expression of stemness markers in the LUSC and LUAD cohorts from TCGA. The Spearman correlation analysis revealed a positive correlation between *USP51* and *CD44*, *SOX2*, *NANOG*, and *OCT4* (as shown in Fig. 2B–E), except for C-MYC and EPCAM, which did not exhibit such a correlation (Fig. 2F, G). Considering these results collectively, we identified a general trend of positive correlations between *USP51* expression and stemness marker expression in patients with NSCLC.

### USP51 knockdown attenuates the stemness of NSCLC cells

**Fig. 3:**
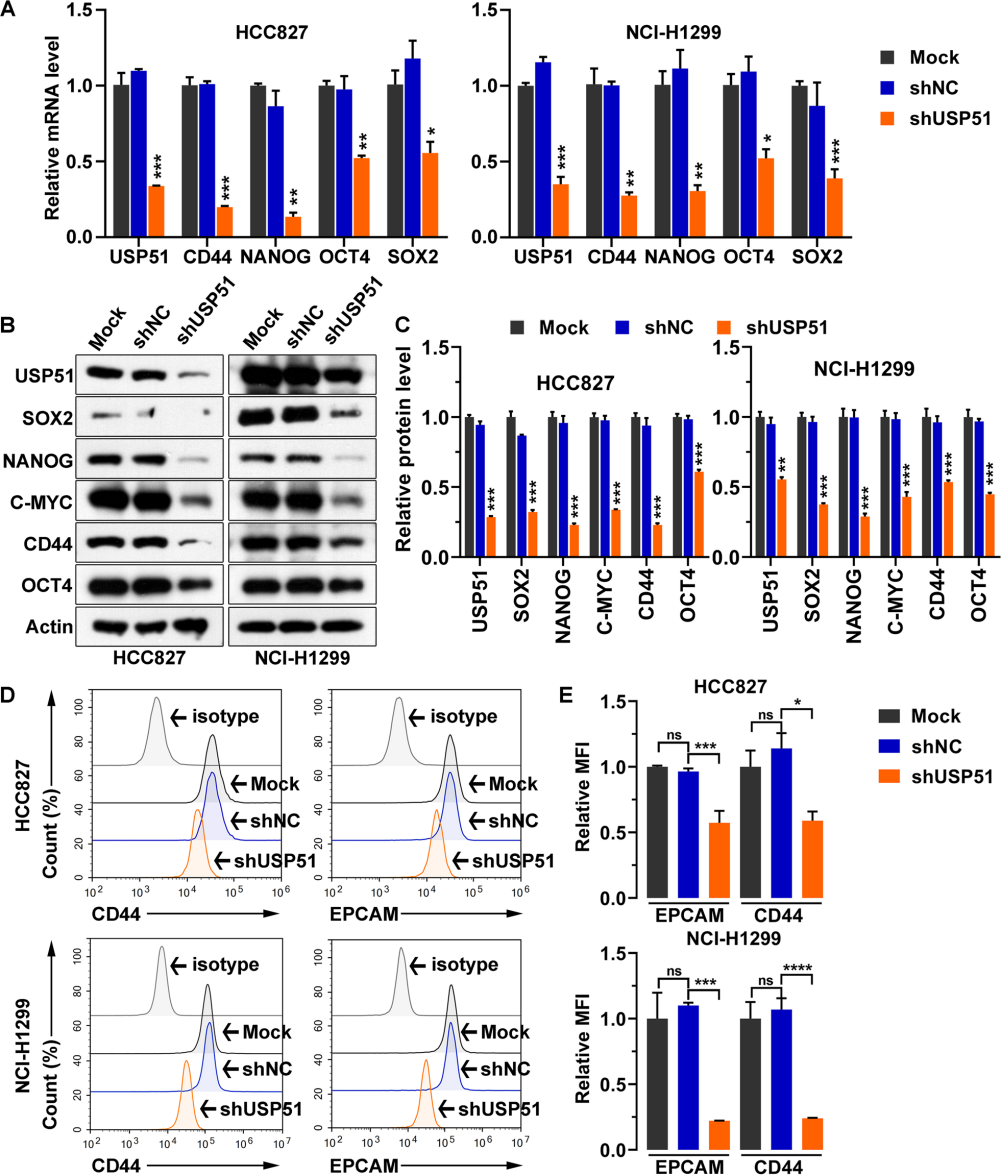
USP51 knockdown mitigated the stemness of HCC827 and NCI-H1299 NSCLC cells. **A** RT‒qPCR quantification of the expression of USP51 and the indicated stemness markers upon USP51 knockdown. **B**, **C** Western blotting (**B**) and quantification (**C**) of USP51 levels in USP51-depleted cells. **D**, **E** Representative images (**D**) and quantification (**E**) of cell surface CD44 and EPCAM expression upon USP51 knockdown

**Fig. 4:**
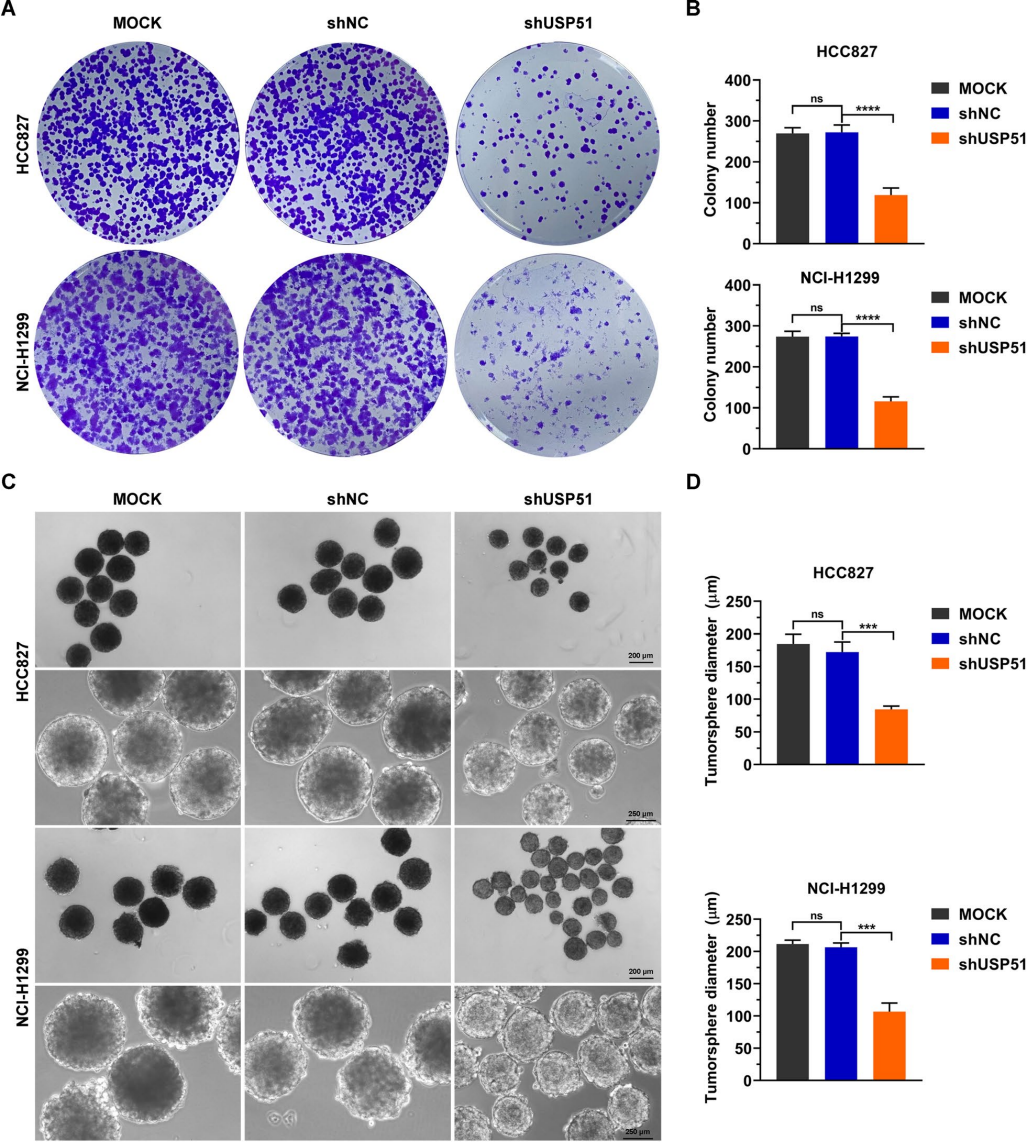
USP51 depletion diminished NSCLC cell stemness. **A**, **B** Colony formation assay of HCC827 and NCI-H1299 cells with USP51 depletion. **C**, **D** Tumor sphere formation assay of HCC827 and NCI-H1299 cells with USP51 knockdown

As cancer stem cells are a key population of cancer cells that result in tumor progression and recurrence [7], we determined the impact of USP51 on NSCLC cell stemness. USP51 was depleted in two NSCLC cell lines, HCC827 and NCI-H1299, by transducing the cells with shRNA. Compared with nontransduced cells (Mock) or cells transduced with a nontargeting shRNA (shNC), USP51 shRNA-transduced cells exhibited marked downregulation of *USP51* mRNA and protein expression (Fig. 3A). Moreover, the expression of stemness markers, including CD44, NANOG, OCT4, SOX2 and C-MYC, was suppressed upon USP51 depletion (Fig. 3B, C). Consistent with these results, USP51 depletion greatly reduced the cell surface levels of two stemness markers, EPCAM and CD44 (Fig. 3D, E). Furthermore, the results of the colony formation assay demonstrated that USP51 depletion led to the formation of fewer colonies by NSCLC cells (Fig. 4A, B). Furthermore, the tumor sphere formation ability of NSCLC cells was decreased upon USP51 knockdown, as determined by measurement of the tumor sphere diameter (Fig. 4C, D). Taken together, these findings indicate that USP51 knockdown dramatically diminishes the stemness of NSCLC cells.

### USP51 deubiquitinates TWIST1

**Fig. 5:**
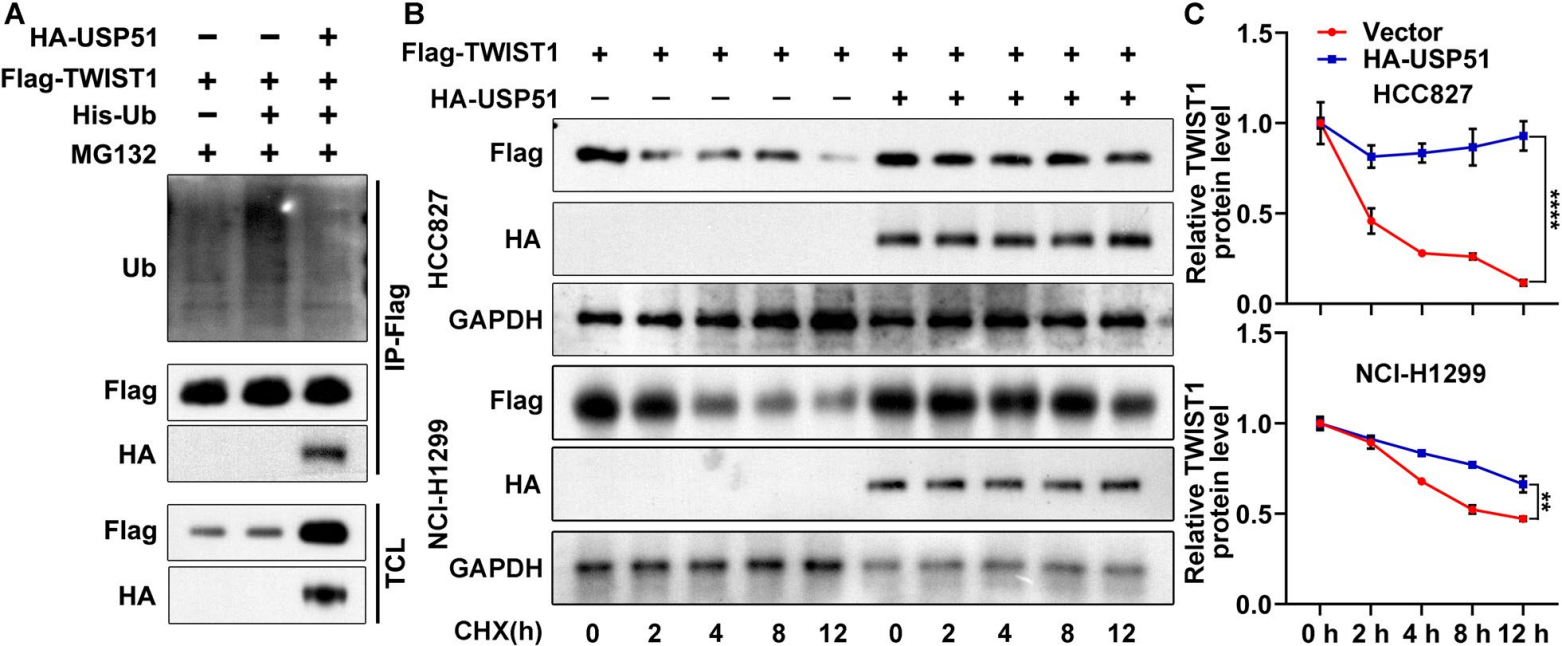
USP51 interacts with TWIST1 and mitigates its polyubiquitination. **A** Co-IP assay to check the interaction between TWIST1 and USP51 and the effect of ectopic USP51 expression on TWIST1 polyubiquitination in HCC827 cells. **B**, **C** Cycloheximide chase time-course assay to determine the stability of TWIST1 upon USP51 overexpression

Given that the TWIST1 protein has been connected to stemness maintenance [17, 18] and that USP51 is a DUB that binds to its targets to modulate their ubiquitination and degradation, we checked whether USP51 affects TWIST1 stability. USP51 interacted with TWIST1, and the ectopic USP51 expression decreased the polyubiquitination of TWIST1 in HCC827 cells in the presence of MG132 (Sigma, M7449), a specific inhibitor of proteasome activity (Fig. 5A). The results of the cycloheximide (CHX) chase timecourse assay suggested that the TWIST1 protein half-life was increased when USP51 was coexpressed with TWIST1 in HCC827 and NCI-H1299 cells (Fig. 5B, C). In conclusion, USP51 interacted with and stabilized TWIST1 by suppressing its polyubiquitination and degradation.

### Increasing TWIST1 expression alleviates the inhibitory effects of USP51 depletion on the proliferation and stemness of NSCLC cells

**Fig. 6:**
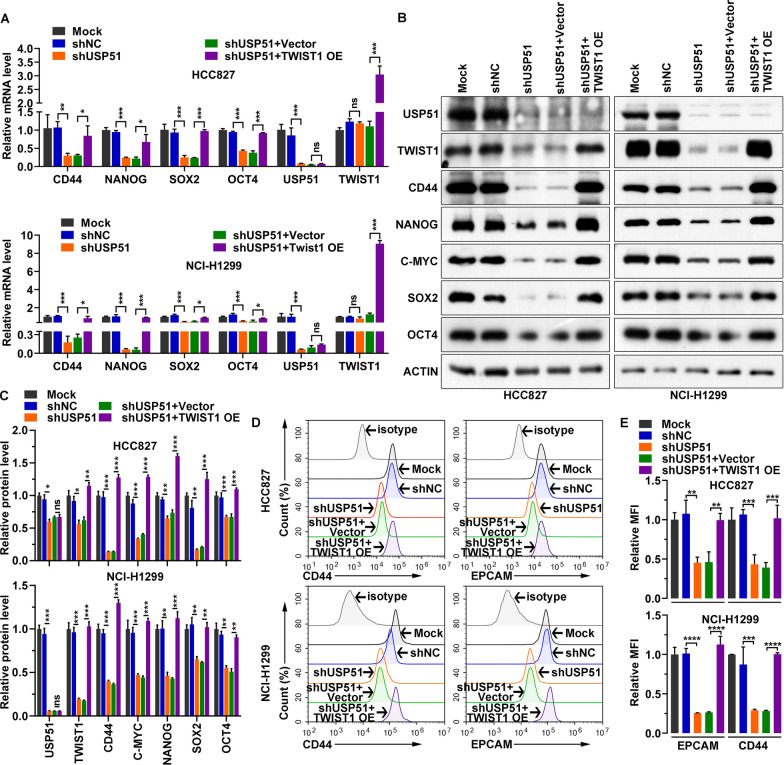
TWIST1alleviates the decrease in stemness marker expression caused by USP51 knockdown in NSCLC cells. **A** RT‒qPCR quantification of the expression of *USP51* and the indicated stemness markers upon USP51 knockdown and TWIST1 overexpression in HCC827 and NCI-H1299 cells. **B**, **C** Western blot analysis of USP51, TWIST1 and stemness marker expression in HCC827 and NCI-H1299 cells upon USP51 knockdown and TWIST1 overexpression. **D**, **E** Representative images (**D**) and quantification (**E**) of cell surface CD44 and EPCAM expression in HCC827 and NCI-H1299 cells upon USP51 knockdown and TWIST1 overexpression

**Fig. 7:**
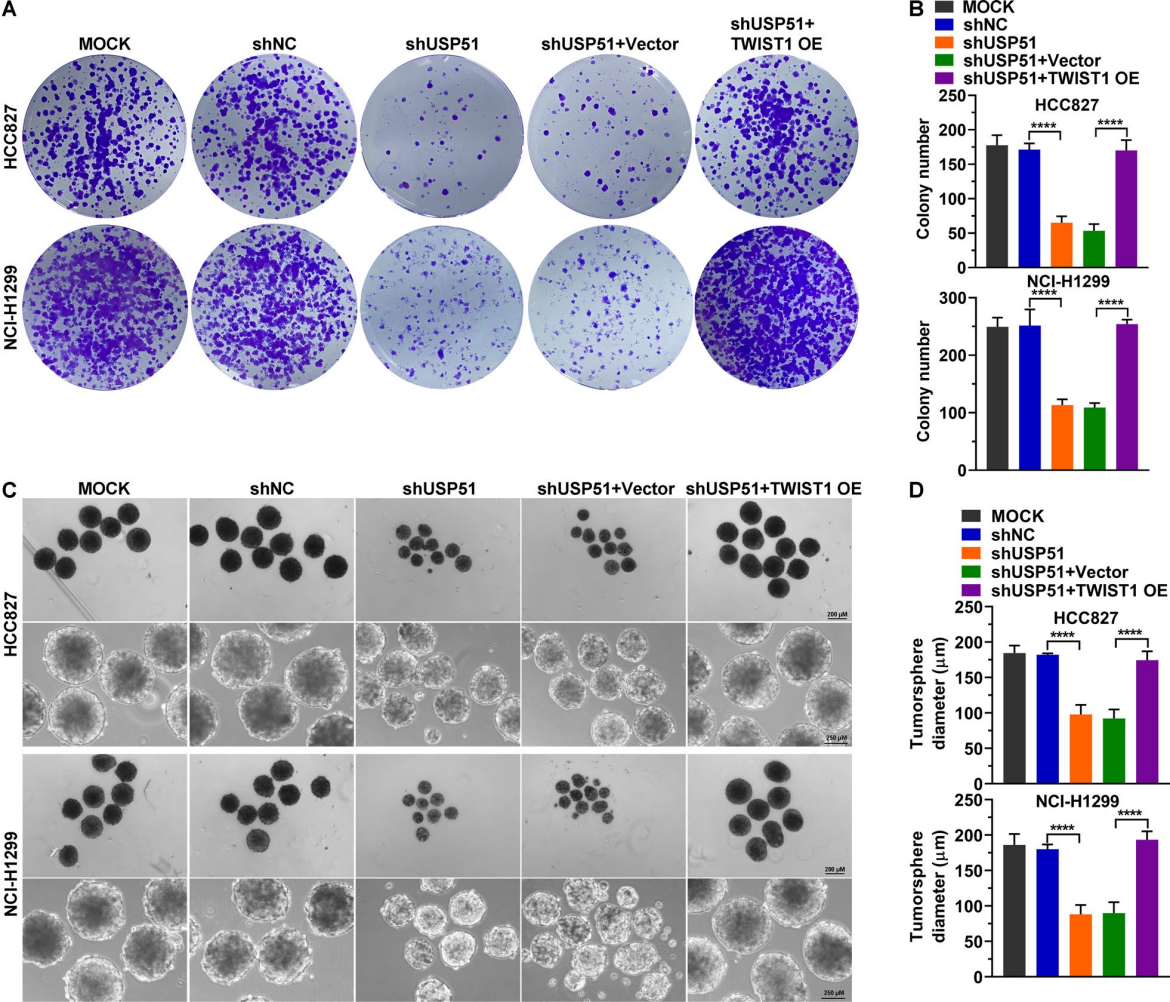
TWIST1 restored the colony and tumor sphere formation, which were reduced by USP51 depletion. **A**, **B** Colony formation activity of NSCLC cells upon USP51 knockdown and TWIST1 overexpression. (C, D) Tumor sphere assay of HCC827 and NCI-H1299 cells upon USP51 knockdown and TWIST1 overexpression

To further determine whether TWIST1 is required for the stemness-enhancing effects of USP51 on NSCLC cells, we ectopically expressed TWIST1 in USP51depleted cells (Fig. 6A–C). Ectopic TWIST1 expression restored the expression of stemness markers CD44, NANOG, SOX2, OCT4, and C-MYC, that was attenuated by USP51 depletion (Fig. 6A–C). Similarly, the decrease in the cell surface levels of CD44 and EPCAM resulting from USP51 knockdown was reversed when TWIST1 was re-expressed in NSCLC cells (Fig. 6D, E). Additionally, re-expression of TWIST1 in NSCLC cells alleviated the USP51 knockdown-mediated inhibition of colony formation and stemness in NSCLC cells (Fig. 7A–D). Overall, these results indicate that overexpression of TWIST1 attenuates the inhibitory effects of USP51 depletion on the proliferation and stemness of NSCLC cells.

### Absence of USP51 expression reduces the tumorigenic ability of NSCLC cells in mice

**Fig. 8:**
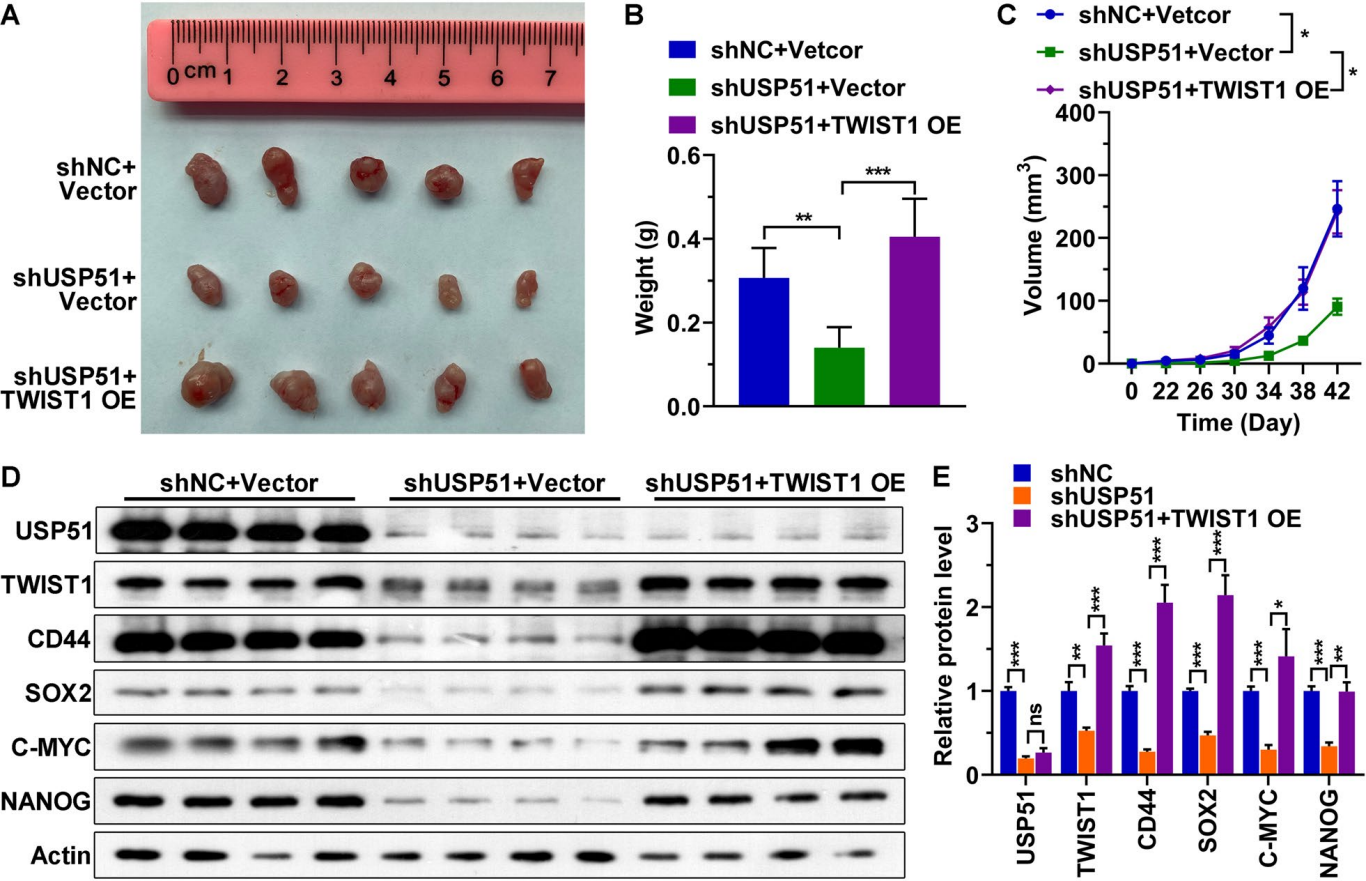
USP51 knockdown reduced the tumorigenic ability of NSCLC cells in mice. **A**–**C** Representative images (**A**) and weights (**B**) and volumes (**C**) of tumors formed by HCC827 cells upon USP51 knockdown and TWIST1 overexpression. **D**, **E** Representative Western blot images (**D**) and quantification (**E**) of USP51, TWIST1 and stemness marker expression in tumors formed by HCC827 cells with USP51 knockdown and TWIST1 overexpression

To confirm our in vitro results, HCC827 cells were subcutaneously injected into nude mice. Dramatically decreases in the tumor weight and volume were observed upon USP51 depletion, and increasing TWIST1 expression reversed the effect of USP51 knockdown on decreasing the tumor weight and volume (Fig. 8A–C). Similar to our observations in in vitro-cultured cells, analysis of lysates from mouse-derived tumors indicated that overexpression of TWIST1 alleviated the decreases in the expression of stemness markers caused by USP51 knockdown in the tumor cells (Fig. 8D, E). In summary, USP51 enhances the tumorigenic ability of NSCLC cells in mice by stabilizing TWIST1.

## Discussion

USP51 belongs to the ubiquitin-specific protease family, which is the largest group of DUBs [19]. USP51 is a DUB that antagonizes RNF168-mediated ubiquitination at DNA double-strand break sites, which promotes aberrant DNA repair and destabilizes the genome [20]. A recently published study demonstrated that USP51 is overexpressed in metastatic LUAD [21]. USP51 was first shown to act as a cancer promoter by targeting ZEB1 for deubiquitination in breast cancer [22]. Similarly, ZEB1 was validated as a target of USP51 in NSCLC, and CDK4/6 was found to activate USP51 [21]. The potentiating effect of USP51 on NSCLC metastasis was shown to result from its targeting of ZEB1 for stabilization [21]. However, whether USP51 is involved in cancer cell stemness has not yet been investigated. In the present study, we discovered positive correlations between *USP51* expression and stemness marker expression in NSCLC using bioinformatics analysis. In the absence of USP51, NSCLC cells showed decreased expression of stemness markers and attenuated growth and tumor sphere formation. Subsequently, TWIST1 was validated as a target of USP51 for deubiquitination, and rescue experiments confirmed the importance of TWIST1 in the functions of USP51 in NSCLC stemness regulation. Collectively, our findings confirmed that the USP51/TWIST1 axis is an underlying mediator of NSCLC cell stemness, supporting the need to develop specific drugs for targeting USP51.

*TWIST1* is a transcription factor that induces epithelial-to-mesenchymal transition (EMT) [23]. Cancer cells undergoing EMT are highly invasive and acquire stem cell properties [24]. TWIST1 is modulated at a variety of levels, including the posttranslational level, by DUBs [25]. DUB3 can deubiquitinate and stabilize TWIST1 upon stimulation by Interleukin 6 (IL-6) [26–28]. Moreover, TWIST1 is deubiquitinated by USP4 in NSCLC cells, resulting in an increased stemness of NSCLC cells [18]. Here, we identified USP51 as a novel modulator of TWIST1 expression and linked TWIST1 with the stemness-promoting effect of USP51. In addition, we found that knockdown of USP51 in NSCLC cells did not reduce the *TWIST1* mRNA level, but did reduce the TWIST1 protein level, indicating that USP51 is not involved in the transcriptional and pretranslational regulation of TWIST1. Given that a DUB may have multiple targets and that a protein can be targeted by various DUBs, we cannot exclude the possibility that other DUBs may also control the ubiquitination-deubiquitination process of TWIST1, thereby fine-tuning the TWIST1 protein level under certain conditions. To systematically identify DUBs targeting TWIST1, a method for screening TWIST1-interacting DUBs can be performed by overexpressing the DUB library in cells. Additionally, the protein interactome of TWIST1 can be used to reveal more DUBs and other interacting modulators of TWIST1. Such analyses can help us better understand the regulatory network of TWIST1, which can further aid in targeting TWIST1 in a more effective manner.

Cancer stem cells are a key population of cancer cells that despite their low abundance, control the reseeding of new tumors [29]. In this study, we found that the expression of USP51 was positively correlated with the expression of stemness markers in NSCLC. Knockdown of USP51 decreased the expression of CD44, NANOG, SOX2, OCT4, C-MYC, and EPCAM. Therefore, we consider that USP51 is associated with cancer cell stemness and cancer progression in patients with NSCLC. However, further clinical evidence is required to support this conclusion.

## Conclusions

**Fig. 9:**
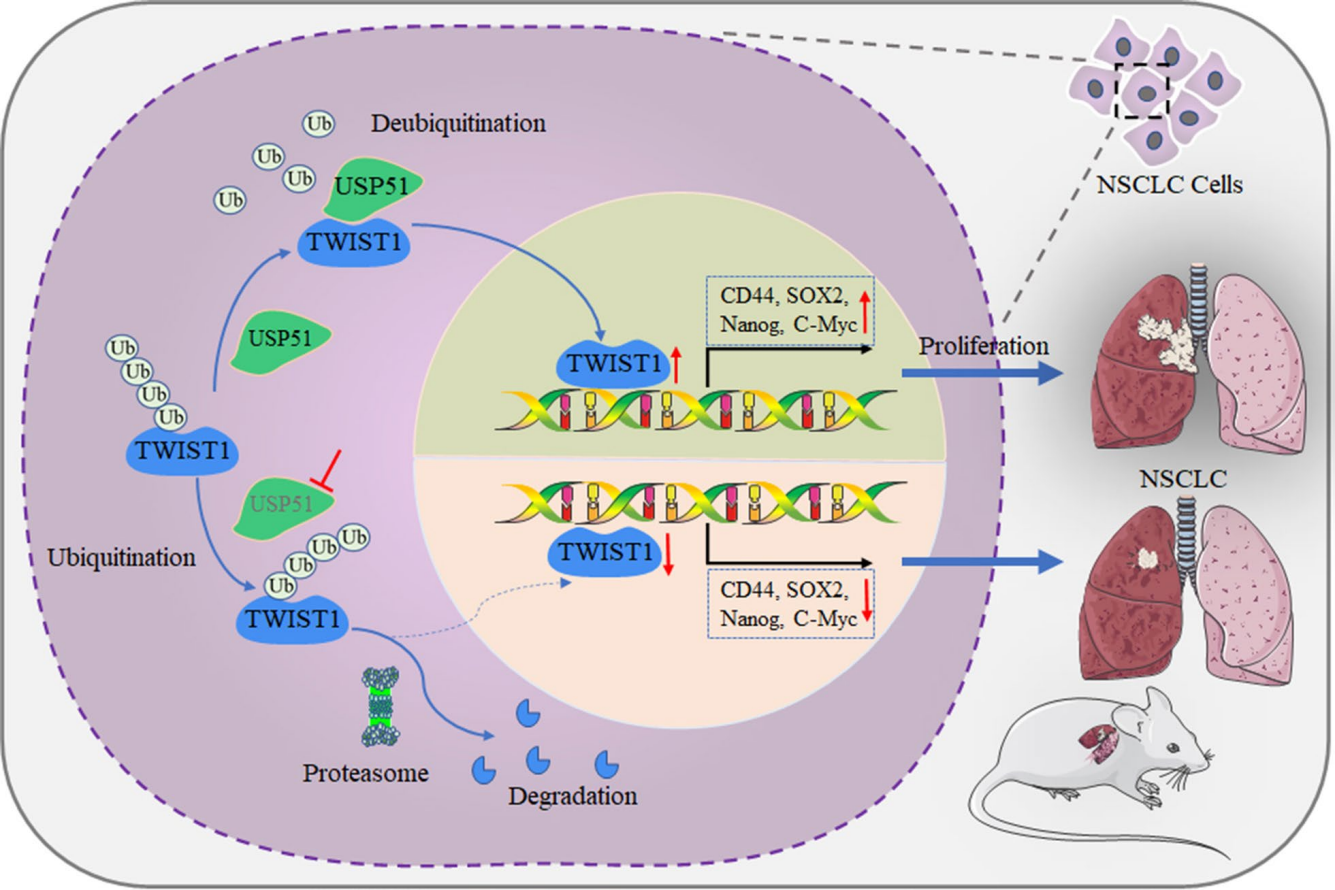
Schematic illustration of the role of USP51 and TWIST1 in NSCLC cell

Taken together, our investigation revealed that USP51 promotes the stabilization of the TWIST1 protein by deubiquitinating TWIST1, which enhances the stemness of NSCLC cells, leading to NSCLC tumor proliferation. Upon depletion of USP51, TWIST1 is polyubiquitinated and subsequently degraded by the proteasome, resulting in a decrease in NSCLC cell stemness and in vivo inhibition of tumor growth (Fig. 9); these finding suggest that USP51 could be a candidate therapeutic target for NSCLC.

## Supplementary Information

**Additional file 1: Figure S1**. Analysis of data from GEO.The expression levels of TWIST1and USP51in lung cancer and normal tissues were compared.The expression levels of TWIST1 and USP51 were compared in patients of different ages.The expression levels of TWIST1 and USP51 were compared in patients at different stages. **Table S1**. Primers for plasmid construction and real-time quantitative PCR. **Table S2**. Antibodies for western blotting.

## Data Availability

All data associated with this study are available from the corresponding author upon request.

## Abbreviations

BCA: Bicinchoninic acid
bFGF: Basic fibroblast growth factor
DMEM: Dulbecco’s modified Eagle’s medium
DUB: Deubiquitinase
EGF: Epidermal Growth Factor
EGFR: Epidermal Growth Factor Receptor
EMT: Epithelial-to-mesenchymal transition
EPCAM: Epithelial Cellular Adhesion Molecule
FBS: Fetal bovine serum
IL-6: Interleukin 6
Mock: Non-transduced cells
NSCLC: Non-small-cell lung carcinoma
RT-qPCR: Real-time quantitative PCR
SDS-PAGE: Sodium Dodecyl Sulfate-Polyacrylamide Gel Electrophoresis
TCGA: The Cancer Genome Atlas
ZEB1: Zinc Finger E-Box Binding Homeobox 1
