# Supplementary material for: USP51 promotes non-small cell lung carcinoma cell stemness by deubiquitinating TWIST1
Source: J Transl Med. 2023 Jul 8;21:453. doi: 10.1186/s12967-023-04304-2 (PMC10329790; doi:10.1186/s12967-023-04304-2)
Supplement: Supplementary file 1 — Additional file 1: Figure S1. Analysis of data from GEO.The expression levels of TWIST1and USP51in lung cancer and normal tissues were compared.The expression levels of TWIST1 and USP51 were compared in patients of different ages.The expression levels of TWIST1 and USP51 were compared in patients at different stages. Table S1. Primers for plasmid construction and real-time quantitative PCR. Table S2. Antibodies for western blotting. [file 12967_2023_4304_MOESM1_ESM.docx]

**Additional file 1:**


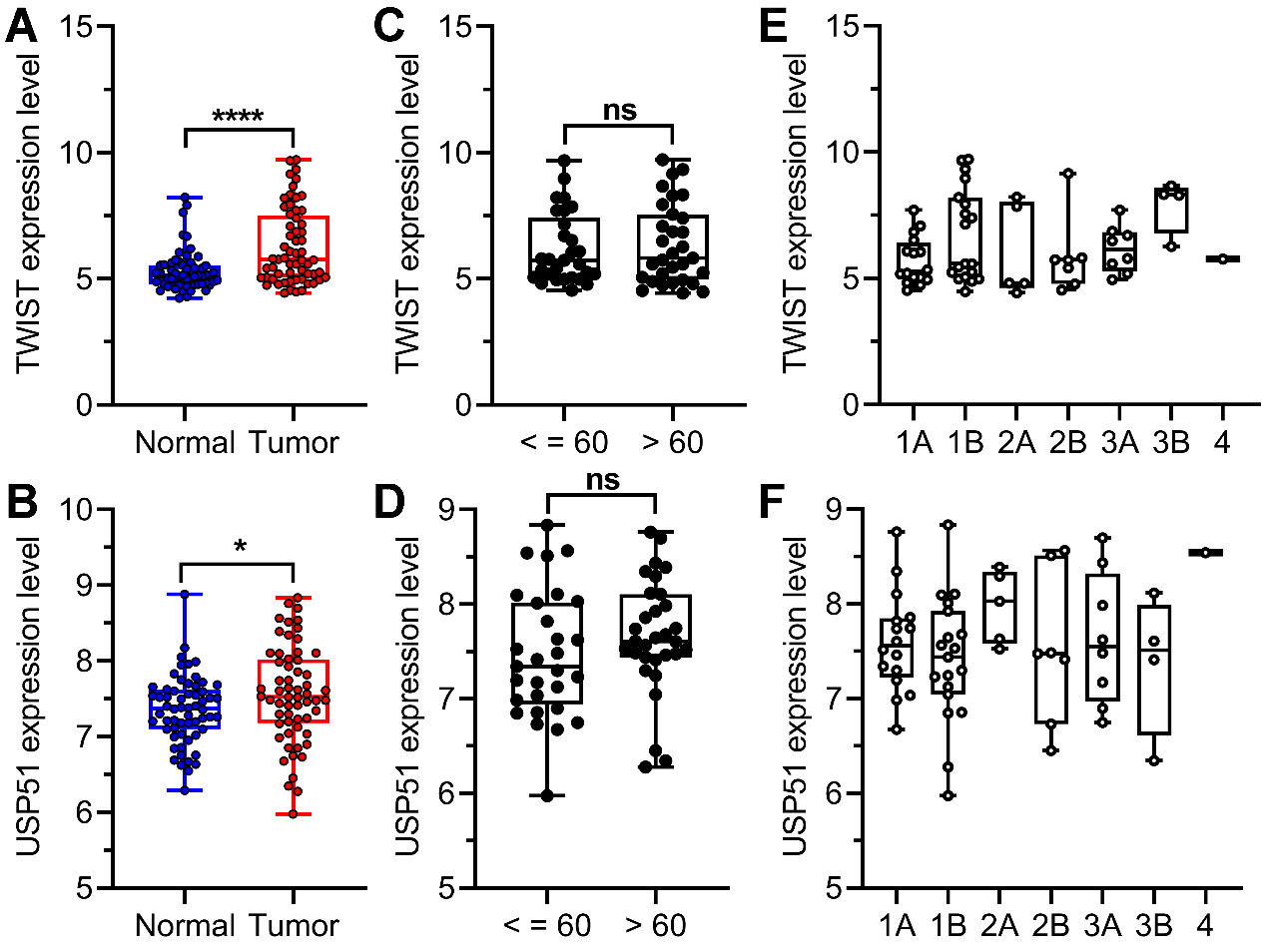


**Fig. S1: Analysis of data from GEO.** (A, B) The expression levels of TWIST1 (A) and USP51 (B) in lung cancer and normal tissues were compared. (C and D) The expression levels of TWIST1 and USP51 were compared in patients of different ages. (E and F) The expression levels of TWIST1 and USP51 were compared in patients at different stages.

**Table S1. Primers for plasmid construction and real-time quantitative PCR**

| Classification | Primer name | Sequence (5’-3’) |
| --- | --- | --- |
| plasmid construction | pLKO.1-USP51 forward | CCGGGCCTGCAATCAGATGTCACATCTCGAGATGTGACATCTGATTGCAGGCTTTTT |
|  | pLKO.1-USP51 shUSP51 reverse | AATTAAAAAGCCTGCAATCAGATGTCACATCTCGAGATGTGACATCTGATTGCAGGC |
|  | pLV-EF1a-HA-USP51 forward | CTAGAGAATTCGGATCCATGGCCCAGGTTCGAGAAAC |
|  | pLV-EF1a-HA-USP51 reverse | GCTTCCATGGCTCGAGCTAGTCTTTCTCTAGACCCTG |
|  | pLV-EF1a-Flag-TWIST1 forward | GAAATGTACAAGGAATTATGATGCAGGACGTGTCCAG |
|  | pLV-EF1a-Flag-TWIST1 reverse | ATTATCTAGGGATCCTTAGTGGGACGCGGACATGGACC |
| Real-time quantitative PCR | RNA18SN5 forward | AGGCGCGCAAATTACCCAATCC |
|  | RNA18SN5 reverse | GCCCTCCAATTGTTCCTCGTTAAG |
|  | USP51 forward | GTTGCCAAAGCTACCAGGAGTC |
|  | USP51 reverse | GCCTACATGCTCAAACCGCTTG |
|  | TWIST1 forward | GCCAGGTACATCGACTTCCTCT |
|  | TWIST1 reverse | TCCATCCTCCAGACCGAGAAGG |
|  | CD44 forward | CCAGAAGGAACAGTGGTTTGGC |
|  | CD44 reverse | ACTGTCCTCTGGGCTTGGTGTT |
|  | NANOG forward | CTCCAACATCCTGAACCTCAGC |
|  | NANOG reverse | CGTCACACCATTGCTATTCTTCG |
|  | OCT4 forward | CCTGAAGCAGAAGAGGATCACC |
|  | OCT4 reverse | AAAGCGGCAGATGGTCGTTTGG |
|  | SOX2 forward | GCTACAGCATGATGCAGGACCA |
|  | SOX2 reverse | TCTGCGAGCTGGTCATGGAGTT |

**Table S2. Antibodies for western blotting**

| Classification | Antibodies | Provider | Catalog number | Dilution |
| --- | --- | --- | --- | --- |
| Primary antibody | Actin | Proteintech, Wuhan, China | 20536-1-AP | 1:3000 |
|  | TWIST1 | Proteintech, Wuhan, China | 25465-1-AP | 1:1500 |
|  | USP51 | Abcam, Shanghai, China | ab121147 | 1:3000 |
|  | CD44 | Proteintech, Wuhan, China | 15675-1-AP | 1:3000 |
|  | SOX2 | Proteintech, Wuhan, China | 66411-1-Ig | 1:3000 |
|  | C-MYC | Proteintech, Wuhan, China | 16286-1-AP | 1:4000 |
|  | NANOG | Proteintech, Wuhan, China | 14295-1-AP | 1:1000 |
|  | OCT4 | Proteintech, Wuhan, China | 11263-1-AP | 1:1000 |
|  | ubiquitin | Proteintech, Wuhan, China | 10201-2-AP | 1:1000 |
|  | Tag-DYKDDDDK | immunoway, SuZhou, China | YG0004 | 1:1000 |
|  | Tag-HA | Proteintech, Wuhan, China | 51064-2-AP | 1:5000 |
| Secondary antibody | horseradish peroxidase-conjugated goat anti-mouse IgG | Proteintech, Wuhan, China | SA00001-1 | 1:10000 |
|  | horseradish peroxidase-conjugated goat anti-rabbit IgG | Proteintech, Wuhan, China | SA00001-2 | 1:10000 |
